# Sex-differences in incidence of hospitalizations and in hospital mortality of community-acquired pneumonia among children in Spain: a population-based study

**DOI:** 10.1007/s00431-022-04478-9

**Published:** 2022-04-25

**Authors:** Javier de-Miguel-Díez, Ana López-de-Andrés, Valentín Hernández-Barrera, José M. de-Miguel-Yanes, David Carabantes-Alarcón, Zichen Ji, Jose J. Zamorano-Leon, Rodrigo Jiménez-García

**Affiliations:** 1 Respiratory Department, Hospital General Universitario Gregorio Marañón, Instituto de Investigación Sanitaria Gregorio Marañón (IiSGM), Faculty of Medicine, Universidad Complutense de Madrid, 28040 Madrid, Spain; 2 Department of Public Health & Maternal and Child Health, Faculty of Medicine, Universidad Complutense de Madrid, 28040 Madrid, Spain; 3 Preventive Medicine and Public Health Teaching and Research Unit, Health Sciences Faculty, Universidad Rey Juan Carlos, 28922 Madrid, Spain; 4 Internal Medicine Department, Hospital General Universitario Gregorio Marañón, Instituto de Investigación Sanitaria Gregorio Marañón (IiSGM), Faculty of Medicine, Universidad Complutense de Madrid, 28040 Madrid, Spain

**Keywords:** Community-acquired pneumonia, Children, Hospital admissions, Incidence, In-hospital mortality, Sex differences

## Abstract

The aim of this study is to examine trends from 2016 to 2019 in the incidence of hospitalizations and in-hospital mortality (IHM) of subjects < 18 years with community-acquired pneumonia (CAP), assessing possible sex differences. We used Spanish national hospital discharge data to select all patients < 18 years with CAP. We identified 43,511 children (53% boys) hospitalized with CAP. The incidence of CAP was significantly higher in boys than in girls, with an age-adjusted incidence rate ratio of 1.05 (95%CI 1.03–1.07) for boys compared to girls, and rose from 126 per 100,000 children in 2016 to 131 in 2019 (*p* < 0.0001). There were no sex differences in isolated pathogens, comorbidities, length of hospital stay, or IHM. Variables independently associated with IHM were age 10 to 17 years, congenital heart disease, neurological diseases, and use of invasive mechanical ventilation. Asthma was a protective factor for IHM among girls.

*Conclusion*: The incidence of hospital admissions for CAP was higher among boys than among girls and rose significantly from 2016 to 2019. There were no sex differences in hospital outcomes. Age 10 to 17 years, congenital heart disease, neurological diseases, and use of mechanical ventilation were risk factors for IHM in both sexes, while asthma was a protective factor among girls. No differences were found in IHM over time.

**Table:** 

**What is Known:** *• Community-acquired pneumonia is one of the most common reasons for hospitalizations among children.* *• There are few studies that allow to know the evolution of community-acquired pneumonia in children.*	
**What is New:** *• Incidence of hospital admissions for community-acquired pneumonia was higher in boys than girls and it rose significantly from 2016 to 2019.* *• Age 10 to 17 years, congenital heart disease, neurological diseases and use of mechanical ventilation were risk factors for in-hospital mortality in both sexes.*

**Supplementary information:**

The online version contains supplementary material available at 10.1007/s00431-022-04478-9.

## Introduction

Community-acquired pneumonia (CAP) has long been recognized as one of the most common causes of hospitalization and a worldwide leading cause of morbidity and mortality among children [1–4].

Many pathogens are responsible for CAP in children, including *Streptococcus pneumonia* and *Haemophilus influenzae*. Several viral pathogens have also been acknowledged as important pathogens for CAP, including influenza virus, respiratory syncytial virus, and adenoviruses [5]. In any case, it must be considered that conventional microbiological studies can identify the etiology in only a limited number of patients [6, 7].

In recent years, pneumonia-associated mortality has decreased in both developing and developed countries as a consequence of improvements in medical care, antiviral and antibacterial treatments, and the effect of vaccines as a preventive tool [8, 9]. In this way, the introduction of pneumococcal conjugate vaccines (PCV) has had a great impact on hospital admissions and mortality from CAP in both low- and high-income countries [10–13]. It is important to remember that pneumococcal vaccination was started in Spain in 2002, with PCV7 that was used until 2010. PCV10 was introduced in 2009 and used until 2014, and PCV13 was introduced in 2010 and is the only vaccine currently used [14].

Epidemiological data on children with CAP in Spain are scarce. Jiménez-Trujillo et al. [14] examined the Spanish National Hospital Database (SNHDD) from 2001 to 2014 and showed that CAP incidence rates decreased significantly among children < 2 years of age. At the same time, in-hospital mortality (IHM) fell significantly in children and adolescents. However, there are few studies that allow us to know the temporal evolution after this period [7].

In this study, we used national discharge data to (a) examine trends from 2016 to 2019 in incidence and in-hospital mortality of CAP subjects aged under 18 years, assessing possible sex differences; (b) describe and compare patient comorbidities, therapeutic and diagnostic procedures, and pathogen isolations between boys and girls; (c) identify factors independently associated with IHM after CAP for both sexes; and (d) compare the results for the period 2016–2019 with those for the period 2001–2014.

## Materials and methods

### Study design and data source

We designed an observational, retrospective epidemiological study. The data source is the SNHDD. Using the International Classification of Diseases 10th edition (ICD-10), up to a maximum of 20 discharge diagnoses and 20 diagnostic or therapeutic procedures performed during the hospital stay can be codified for each hospitalization [15].

### Study population and study variables

We analyzed data collected by the SNHDD from January 1, 2016, to December 31, 2019, for subjects aged ≤ 17 years.

We included those children who had a code for CAP as detailed in Supplementary Table 1. The codes for pneumonia in the ICD-10 are “J12” to “J18”. Additionally, the SNHDD has a present on admission (POA) indicator assigned for each diagnosis code. Only those pneumonia codes in any diagnosis position with a POA indicator of “Yes” were included. Those records with a POA code “No” or “Clinically Undetermined” or “Unknown” were excluded. Additionally, those children with missing data on age sex or length of hospital stay were not included in the study population. The initial number of children identified with CAP was 43,774. Those records with missing data for age (*n* = 11; < 0.1%), sex (*n* = 7; < 0.1%), or duration of the hospitalization (*n* = 245; 0.6%) were excluded with no imputation of missing data.

The study population was stratified by sex and age groups (< 2 years, 2–4 years, 5–9 years, and 10–17 years) as in the previous work by Jimenez Trujillo et al. [14].

The main study variables were the incidence of CAP, the IHM, and the length of hospital stay (LOHS). Incidences were estimated using data obtained by the Spanish National Statistics Institute that provided the population samples by sex and age groups for each of the study years [16]. IHM is defined by the proportion of children who died during hospital admission in each year of study.

Study covariates included the presence of the following diseases and conditions, irrespective of their position on the diagnosis coding list: asthma, congenital heart disease, Down syndrome and other chromosome anomalies, neurological disease, and diabetes. Finally, we identified procedures (invasive mechanical ventilation, noninvasive mechanical ventilation, and thoracocentesis) and lab-confirmed pathogens documented during hospitalizations for CAP (*Streptococcus pneumoniae*, influenza virus, and “other virus”). The ICD10 codes used for variable definitions are shown in Supplementary Table 1.

### Statistical analysis

To assess time trends, the incidence rates of CAP were calculated per 100,000 boys and girls and according to age. A time trend analysis was performed using Poisson regression, adjusted by age and sex when needed. Incidence rate ratios (IRR), with 95% confidence intervals (95% CI), were the measures of association calculated.

We provided a descriptive statistical analysis with total frequencies and proportions for qualitative variables and means with standard deviations (SD) or medians with interquartile ranges (IQR) for quantitative variables.

The changes in proportions from 2016 to 2019 were analyzed with the χ^2^ test for linear trends, means with ANOVA, and medians with the Kruskal–Wallis test, as appropriate.

Finally, to identify variables independently associated with IHM, we performed three logistic regression analyses for boys, girls and both sexes, following the recommendations of Hosmer et al. [17]. The variables included in the models were those with a significant association in the univariate analysis. Estimates were odds ratios (OR) with their 95% CI.

Statistical analyses were performed using Stata version 14 (Stata, College Station, Texas, USA). Statistical significance was set at *p* < 0.05 (2-tailed).

### Ethical aspects

Since SNHDD data have no personal identifiers, this study was considered exempt from review by an Ethics Committee according to Spanish legislation.

## Results

From 2016 to 2019, a total of 43,511 patients aged under 18 years were discharged from Spanish hospitals with CAP. The proportion of boys was 53% (22,942). By age group, the youngest children (< 2 years) represented 37% of hospitalizations (39% for boys vs. 35% for girls). The numbers of boys and girls decreased with age, with the lowest figures for those aged 10 to 17 years.

### Trends from 2016 to 2019 in the incidence of CAP

**Fig. 1:**
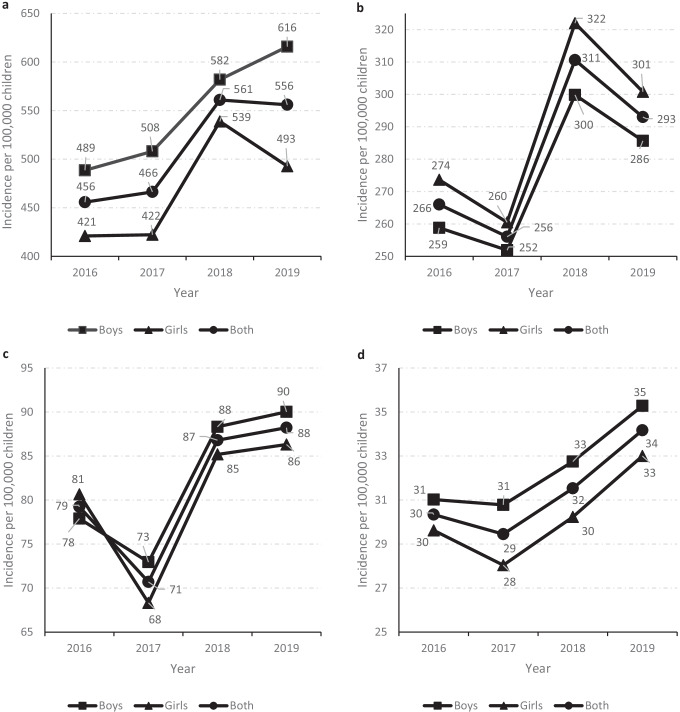
Incidence of hospital discharges among children with community-acquired pneumonia in Spain from 2016 to 2019 according to sex and age group. **a** Children under 2 years. **b** Children aged 2 to 4 years. **c** Children aged 5 to 9 years. **d** Children aged 10 to 17 years

The overall incidence of CAP increased from 126 per 100,000 children in 2016 to 131 cases per 100,000 children in 2019 (*p* < 0.001). Over the entire period, the incidence was highest among children < 2 years of age (508 cases per 100,000 children), decreasing with increasing age to only 31 cases per 100,000 children among those aged 10–17 years. In all the years analyzed and for all age groups, incidences were significantly higher in boys than in girls and rose significantly from 2016 to 2019 (Fig. 1). Overall, the age-adjusted incidence rate ratio was 1.05 (95% CI 1.03–1.07) for boys compared to girls.

### Sex differences in hospital discharges with CAP

Table 1 shows the characteristics, comorbidities, diagnostic and therapeutic procedures, isolated pathogens, and in-hospital outcomes among children (< 18 years). Boys were slightly but not significantly younger than girls (3.7 years vs. 3.8 years; *p* = 0.084) as can be seen in Supplementary Table 1.

**Table 1:** Distribution and in-hospital mortality according to demographic characteristics, comorbidities, diagnostic and therapeutic procedures, isolated pathogens, and length of hospital stay among children (< 18 years) discharged with community-acquired pneumonia in Spain from 2016 to 2019

		Distribution	IHM
Sex	Boys	22,942 (53)	73 (0.3)
	Girls	20,569 (47)	75 (0.4)
Age groups	< 2 years, *n* (%)	48 (0.3)	48 (0.3)
	2–4 years, *n* (%)	22 (0.2)	22 (0.2)
	5–9 years, *n* (%)	22 (0.3)	22 (0.28)
	10–17 years, *n* (%)	56 (1.2)	56 (1.2)
Age, mean(SD)		3.75 (3)	7.01 (6)
Asthma, *n* (%)		2463 (5.7)	3 (0.1)
Congenital heart disease, *n* (%)		780 (1.8)	11 (1.4)
Down syndrome and other chromosome anomalies, *n* (%)		914 (2.1)	5 (0.6)
Neurological disease, *n* (%)		1454 (3.3)	61 (4.2)
Diabetes, *n* (%)		66 (0.2)	1 (1.5)
Invasive mechanical ventilation, *n* (%)		648 (1.5)	59 (9.1)
Non-invasive mechanical ventilation, *n* (%)		1410 (3.2)	37 (2.6)
Thoracocentesis, *n* (%)		946 (2.2)	9 (1)
*S. pneumoniae*, *n* (%)		1537 (3.5)	6 (0.4)
Influenza virus, *n* (%)		2098 (4.8)	9 (0.4)
Other virus, *n* (%)		5223 (12)	20 (0.4)
Length of hospital stay, median (IQR)		4 (3)	4 (10.5)
2016, *n* (%)		10,427 (24)	44 (0.4)
2017, *n* (%)		9991 (23)	35 (0.4)
2018, *n* (%)		11,735 (27)	31 (0.3)
2019, *n* (%)		11,358 (26.1)	38 (0.3)
Total, *n* (%)		43,511 (100)	148 (0.3)

*IHM* in-hospital mortality, *SD* standard deviation, *IQR* inter quartile range

The most prevalent chronic condition was asthma, followed by neurological disease, Down syndrome, and other chromosome anomalies and congenital heart disease, with no differences by sex. Diabetes appeared in under 0.4% of children.

Non-invasive mechanical ventilation was used twice as frequently (3.2%) than invasive mechanical ventilation (1.5%), without any sex differences.

Thoracocentesis was conducted in 2.2% of children.

Of the pathogens analyzed, the most isolated were other viruses (≈ 12%), followed by influenza virus (≈ 4.8%) and *S*. *pneumoniae* (≈ 3.5%), and no sex differences were found.

Regarding hospital outcomes, the median LOHS was the same (4 days) for both sexes, and the IHM rates were similar (*p* = 0.406) for boys, 0.3%, and girls, 0.4% (Supplementary Table 2).

### Factors associated with in-hospital mortality among children with CAP

The IHM rates among children with CAP in Spain from 2016 to 2019 according to sociodemographic and clinical characteristics and year are shown in Table 1. The children aged < 2 years showed an IHM of 0.3%, decreasing in the 2–4 years (0.2%) and 5–9 years age groups and increasing up to 1.2% in those aged 10 to 17 years.

Children with neurological diseases had the highest IHM rates (4.2%), followed by children with congenital heart disease (1.4%). However, children with asthma had low IHM rates (0.1%).

Among the procedures studied, receiving invasive mechanical ventilation was associated with very high IHM rates of 9.1%.

Receiving non-invasive mechanical ventilation and thoracocentesis also increased the IHM.

The IHM among those with *S*. *pneumoniae* (0.4%), influenza virus (0.4%), and “other virus” (0.4%) isolations did not differ according to sex.

From 2016 (0.4%) to 2019 (0.3%), the IHM decreased slightly but not significantly (*p* = 0.23). As can be seen in Supplementary Table 3, no significant differences were found in the IHM according to sex for any of the study variables.

The results of the multivariable analysis to identify study variables independently associated with IHM are shown in Table 2. Boys (OR 2.76; 95% CI 1.45–5.27) and girls (OR 1.66; 95% CI 1.07–3.65) aged 10 to 17 years had a significantly higher probability of dying in the hospital when compared with the under 2 years’ age group.

**Table 2:** Multivariable analysis of the factors associated with in hospital mortality among boys and girls hospitalized with community-acquired pneumonia in Spain from 2016 to 2019

		Boys	Girls	Both sexes
		OR CI 95%	OR CI 95%	OR CI 95%
Age groups	< 2 years	Reference	Reference	Reference
	2–4 years	0.4 (0.17–0.9)	0.49(0.25–0.95)	0.46(0.28–0.77)
	5–9 years	0.9(0.43–1.89)	0.52 (0.24–1.13)	0.71 (0.41–1.2)
	10–17 years	2.76 (1.45–5.27)	166 (1.07–3.65)	1.99 (1.25–3.15)
Asthma		NS	0.28 (0.04–0.94)	0.35 (0.11–0.96)
Congenital heart disease		4.27 (1.11–8.99)	3.12 (1.72–7.23)	3.78 (2.07–7.75)
Neurological diseases		8.32 (3.46–13.8)	8.57 (3.13–12.43)	8.93 (4.4–11.17)
Invasive mechanical ventilation		14.43 (8.43–24.69)	18.89 (10.99–32.49)	16.22 (11.09–23.73)

Calculated using multivariable logistic regression models to identify those variables independently associated to IHM

*IHM* In-hospital mortality, *OR* odds ratio, *CI* confidence interval, *NS* not significant

After logistic regression, suffering asthma showed a protective effect for IHM only among girls, while suffering congenital heart disease or neurological diseases multiplied the risk of dying in the hospital by 3 to 4 times and by over 8 times, respectively, in both sexes.

The need for invasive mechanical ventilation was associated with a high risk of IHM among boys (OR 14.43; 95% CI 8.43–24.69) and girls (OR 18.89; 95% CI 10.99–32.49).

### Comparison of the results from 2016 to 2019 with those obtained from 2001 to 2014

In the period 2012–2014, a total of 16,895 boys and 14,847 girls were discharged with a primary diagnosis of CAP [14]; the equivalent figures for the 3-year period 2017–2019 were 13,630 boys and 12,283 girls.

When incidences were compared from 2014 to 2019, significant decreases were found for boys (140 per 100.000 boys to 111 per 100.000 boys; *p* < 0.001) and for girls (129 per 100.000 girls to 103 per 100.000 girls; *p* < 0.001). The incidences obtained in 2019 were lower than those reported for 2014 for all age groups and both sexes [14].

A diagnosis of *S*. *pneumoniae* was codified in 5.8% of children in the year 2012–2014, decreasing significantly to 3.8% for the period 2017–2019 (*p* < 0.001) [14].

The IHM for the period 2012–2014 was 0.3%, similar to the IHM of 0.3% for the period 2017–2019 (*p* = 0.176) [14].

## Discussion

Our study demonstrated that the incidence of hospital admissions for CAP was higher in boys than in girls and rose significantly from 2016 to 2019. Despite the fact that knowledge of sex differences in the incidence of pneumonia is important to establish preventive strategies, few studies have examined this relationship [18]. Similarly, Wiese et al. [19] reported that the rates of pneumonia hospitalizations for boys were consistently higher than those for girls, but only for those < 2 years. In contrast, Naheed et al. [20] demonstrated that girls have more severe pneumonia at the time of admission, and the risk of a fatal outcome is higher in them. Possible mechanisms that may explain these findings, among older children, include hormonal effects [21, 22]. However, the increase in the incidence of pediatric CAP hospitalizations contrasts with the decline reported by other authors in preceding years after the introduction of the PCV vaccine [3, 14, 23, 24] and could reflect changes in the ICD coding system over time or simply the use of less restrictive admission criteria for CAP in the most recent period.

Pneumonia in children with comorbidities is common [25]. Many investigators have described an association between asthma and an increased incidence of invasive pneumococcal disease, but the underlying mechanisms are uncertain [26–29].

Children with neurological diseases often have an increased risk of pneumonia due to respiratory muscle weakness, lack of central drive, or impaired swallowing [30].

Our study revealed that factors associated with IHM in both sexes were age 10 to 17 years, congenital heart disease, neurological diseases, and use of invasive mechanical ventilation. The reason for a greater IHM in older children may be associated with the evolution of concomitant diseases, as previously suggested by other groups [14]. In addition, patients with congenital heart disease are at elevated risk of pneumonia hospitalizations and pneumonia-associated mortality, with the risk further elevated in those with severe affectation and extra-cardiac defects [31]. The presence of neurological diseases is also a risk factor for death during admission for CAP. Millman et al. recently reported that children with neurologic disorders hospitalized with CAP were more likely to be admitted to the intensive care units than those without neurological diseases [32]. Finally, it is likely that the need for assisted ventilation indicates severity, having previously been identified as an independent risk factor for mortality in children admitted with pneumonia [33, 34].

Interestingly, asthma was a protective factor for IHM among girls, in line with previous studies in patients with other respiratory infections [35]. However, it cannot be ruled out that asthmatic children are admitted to the hospital with less severe infections, resulting in an apparent protective effect [14].

The analysis of the temporal trends of the incidence of hospitalizations between 2014 and 2019, using only CAP as the primary diagnosis, suggests that it is decreasing in both sexes and in all age groups, although the IHM does not change. The reduction in incidence may be related to the decrease observed in the isolation of *S*. *pneumoniae* over time, which may in part be due to the increase in pneumococcal vaccination coverage as described in other countries [36–38]. Regarding IHM, one possible reason why reductions were not found is because childhood pneumonia mortality had already been steadily decreasing in children and adolescents in Spain after PCV introduction until 2014 [14]. Another possibility is that it is being encoded differently following the move from ICD9 to ICD10, which occurred in Spain in January 2016. However, Smithee et al. [39] have recently shown that the ICD-10-CM algorithm derived from a validated ICD-9-CM algorithm should not introduce substantial bias for evaluating pneumonia trends in children.

The most frequent primary diagnosis when CAP appeared as a secondary diagnosis was influenza followed by acute bronchiolitis. These findings were consistent with those previously reported in the literature [40, 41].

There are several limitations that should be considered. The most important limitation is the use of ICD-10 codes to retrospectively identify patients with pneumonia, which could be subject to misclassification. In fact, there is a large overlap in the clinical characteristics of CAP, bronchiolitis, and sometimes even asthma. This was also found in our study, as shown in Supplementary Table 4. When CAP was a secondary diagnosis, the second most common primary diagnosis was bronchiolitis. However, previous investigations have found that the ICD coding of CAP has a specificity higher than 80% and a sensitivity below 70% when medical records are used as a reference [42–46]. The lack of sensitivity means that CAP diagnosis using ICD-based administrative data may underestimate the incidence. However, the very high specificity indicates very few false-positives; thus, most children with a code for CAP truly have this disease [42–46]. Second, as we used an administrative database, we did not have enough clinical data to adequately assess the severity of pneumonia, nor did we have information on the pharmacological treatment received. Third, we did not evaluate changes in medical practice, access to health care or, more importantly, changes in the use of diagnosis codes.

Finally, in the SNHDD, it is not possible to identify those children according to the migrant status. Therefore, we cannot assess if this variable affects the trend of pneumonia severity. However, according to the Spanish National Statistics Institute, the proportion of migrants among children aged 0 to 17 years in Spain has remained stable from 2016 to 2019 (9.76% in 2016, 9.58% in 2017, 9.84% in 2018 and 10.39% in 2019) [16]. So, in our opinion, the possible effect of the migrant status on the severity of pneumonia, if it exists, would be of small magnitude.

Despite these limitations, the strengths of our study lie in its large nationally representative population and the use of a standardized methodology, which reduces the chance of selection bias.

In conclusion, we observed that the incidence of hospital admissions for CAP was higher in boys than in girls and rose significantly from 2016 to 2019. There were no sex differences in hospital outcomes. Age 10 to 17 years, congenital heart disease, neurological diseases, and use of mechanical ventilation were risk factors for IHM in both sexes, while asthma was a protective factor among girls.

## Supplementary information

Below is the link to the electronic supplementary material.

Supplementary file1 (DOCX 42 KB)

## Data Availability

According to the contract signed with the Spanish Ministry of Health and Social Services, which provided access to the databases from the Spanish National Hospital Discharge Database, we cannot share the databases with any other investigator, and we must destroy the databases once the investigation has concluded. Consequently, we cannot upload the databases to any public repository. However, any investigator can apply for access to the databases by filling out the questionnaire available at: http://www.msssi.gob.es/estadEstudios/estadisticas/estadisticas/estMinisterio/SolicitudCMBDdocs/Formulario_Peticion_Datos_CMBD.pdf. All other relevant data are included in the paper.

## Abbreviations

95% CI: 95% Confidence intervals
CAP: Community-acquired pneumonia
ICD-10: International Classification of Diseases 10th edition
IHM: In-hospital mortality
IQR: Inter-quartile range
IRR: Incidence rate ratios
LOHS: Length of hospital stay
OR: Odds Ratios
PCV: Pneumococcal conjugate vaccines
POA: Present on Admission
SD: Standard deviations
SNHDD: Spanish National Hospital Database
