# Supplementary material for: Sex-differences in incidence of hospitalizations and in hospital mortality of community-acquired pneumonia among children in Spain: a population-based study
Source: Eur J Pediatr. 2022 Apr 25;181(7):2705–13. doi: 10.1007/s00431-022-04478-9 (PMC9192385; doi:10.1007/s00431-022-04478-9)
Supplement: Supplementary file 1 — Supplementary file1 (DOCX 42 KB) [file 431_2022_4478_MOESM1_ESM.docx]

**Supplementary table 1**. ICD 10 codes for diagnosis and procedures used in this investigation.

| **Diagnosis and procedures** | **ICD10 Codes** |
| --- | --- |
| Community-acquired pneumonia | J12 to J18 as primary diagnosis with a POA indicator of “Y”.  J12 to J18 in any of the secondary diagnosis fields (2-20) and with a POA indicator of “Y”. |
| Asthma | J45.xx |
| Congenital heart disease | Q20-Q24 |
| Down syndrome and other chromosome anomalies | Q90-Q99 |
| Neurological disease | G40.xx, G80.xx |
| Diabetes | E10 |
| Invasive mechanical ventilation | 5A1945Z, 5A1955Z, 5A1935Z |
| Non-invasive mechanical ventilation | 5A09357, 5A09457, 5A09557 |
| Thoracocentesis | 0W9930Z, 0W993ZZ, 0W993ZX, 0W9B30Z, 0W9B3ZZ, 0W9B3ZX |
| *S. Pneumoniae* | J13.xx |
| Influenza virus | J09.X1, J10.00 J10.01, J10.08, J11.0, J11.00, J11.08 |
| Other virus | J12.XX |

**Supplementary table 2**. Characteristics, comorbidities, diagnostic and therapeutic procedures, isolated pathogens, and in-hospital outcomes and among children (<18 years) discharged with community-acquired pneumonia in Spain from 2016 to 2019 according to sex.

|  | | Boys | Girls | P |
| --- | --- | --- | --- | --- |
| Age, mean (SD) | | 3.72(3.09) | 3.79(2.94) | 0.084 |
| Age groups | < 2 years, n(%) | 8950(39.01) | 7233(35.16) | <0.001 |
|  | 2-4 years, n(%) | 7371(32.13) | 7346(35.71) |  |
|  | 5-9 years, n(%) | 4106(17.9) | 3771(18.33) |  |
|  | 10-17 years, n(%) | 2515(10.96) | 2219(10.79) |  |
| Asthma, n(%) | | 1261(5.5) | 1202(5.84) | 0.118 |
| Congenital heart disease, n(%) | | 384(1.67) | 396(1.93) | 0.058 |
| Down syndrome and other chromosome anomalies, n(%) | | 503(2.19) | 411(2) | 0.158 |
| Neurological disease, n(%) | | 755(3.29) | 699(3.4) | 0.534 |
| Diabetes, n(%) | | 30(0.13) | 36(0.18) | 0.236 |
| Invasive mechanical ventilation, n(%) | | 360(1.57) | 288(1.4) | 0.146 |
| Non-invasive mechanical ventilation, n(%) | | 758(3.3) | 652(3.17) | 0.430 |
| Thoracocentesis, n(%) | | 502(2.19) | 444(2.16) | 0.833 |
| *S. pneumoniae*, n(%) | | 817(3.56) | 720(3.5) | 0.732 |
| Influenza virus, n(%) | | 1089(4.75) | 1009(4.91) | 0.440 |
| Other virus, n(%) | | 2675(11.66) | 2548(12.39) | 0.02 |
| Length of hospital stay, median (IQR) | | 4(3) | 4(3) | 0.803 |
| In-hospital mortality, n(%) | | 73(0.32) | 75(0.36) | 0.406 |

P value for the differences between boys and girls. SD: Standard deviation. IQR: Inter quartile range.

**Supplementary Table 3**. In-hospital mortality among boys and girls discharged with community-acquired pneumonia in Spain from 2016 to 2019 according to socio-demographic and clinical characteristics and year.

|  | | Boys | Girls | p |
| --- | --- | --- | --- | --- |
| Age, mean (SD) | | 7.96(6.48) | 6.09(5.81) | 0.067 |
| Age groups | < 2 years, n(%) | 22(0.25) | 26(0.36) | 0.189 |
|  | 2-4 years, n(%) | 8(0.11) | 14(0.19) | 0.204 |
|  | 5-9 years, n(%) | 12(0.29) | 10(0.27) | 0.820 |
|  | 10-17 years, n(%) | 31(1.23) | 25(1.13) | 0.737 |
| Asthma, n(%) | | 2(0.16) | 1(0.08) | 0.598 |
| Congenital heart disease, n(%) | | 7(1.82) | 4(1.01) | 0.343 |
| Down syndrome and other chromosome anomalies, n(%) | | 3(0.6) | 2(0.49) | 0.823 |
| Neurological disease, n(%) | | 30(3.97) | 31(4.43) | 0.661 |
| Diabetes, n(%) | | 0(0) | 1(2.78) | 0.908 |
| Invasive mechanical ventilation, n(%) | | 28(7.78) | 31(10.76) | 0.191 |
| Non-invasive mechanical ventilation, n(%) | | 17(2.24) | 20(3.07) | 0.336 |
| Thoracocentesis, n(%) | | 5(1) | 4(0.9) | 0.880 |
| *S. pneumoniae*, n(%) | | 4(0.49) | 2(0.28) | 0.512 |
| Influenza virus, n(%) | | 5(0.46) | 4(0.4) | 0.826 |
| Other virus, n(%) | | 8(0.3) | 12(0.47) | 0.319 |
| Length of hospital stay, median (IQR) | | 4(9) | 5(11) | 0.519 |
| 2016, n(%) | | 23(0.42) | 21(0.42) | 0.989 |
| 2017, n(%) | | 15(0.28) | 20(0.43) | 0.213 |
| 2018, n(%) | | 17(0.28) | 14(0.25) | 0.745 |
| 2019, n(%) | | 18(0.30) | 20(0.38) | 0.456 |

P value for the differences between boys and girls. SD: Standard deviation. IQR: Inter quartile range.

**Supplementary table 4**. Most common primary diagnosis when community-acquired pneumonia is not codified as a primary diagnosis in children (<18 years) in Spain from 2016 to 2019 according to age group and sex.

| **BOYS** | | | | | | | | | | | | | | |
| --- | --- | --- | --- | --- | --- | --- | --- | --- | --- | --- | --- | --- | --- | --- |
| **< 2 YEARS** | | | **2-4 YEARS** | | | **5-9 YEARS** | | | **10-17 YEARS** | | | **TOTAL** | | |
| **Disease** | **n** | **%** | **Disease** | **n** | **%** | **Disease** | **n** | **%** | **Disease** | **n** | **%** | **Disease** | **n** | **%** |
| Acute bronchiolitis | 937 | 34.1 | Influenza | 327 | 25.8 | Influenza | 118 | 17.2 | Influenza | 85 | 17.6 | Influenza | 1005 | 19.1 |
| Influenza | 481 | 17.5 | Respiratory failure | 171 | 13.5 | Respiratory failure | 117 | 17.1 | Asthma | 66 | 11.8 | Acute bronchiolitis | 956 | 18.2 |
| Respiratory failure | 401 | 14.6 | Other respiratory disorders | 141 | 11.1 | Asthma | 98 | 14.3 | Respiratory failure | 58 | 10.4 | Respiratory failure | 747 | 14.2 |
| Acute bronchitis | 189 | 6.9 | Acute bronchitis | 122 | 9.6 | Other respiratory disorders | 84 | 12.2 | Sepsis | 38 | 6.8 | Other respiratory disorders | 418 | 7.9 |
| Other respiratory disorders | 165 | 6.0 | Asthma | 97 | 7.7 | Acute bronchitis | 44 | 6.4 | Other respiratory disorders | 28 | 5.0 | Acute bronchitis | 361 | 6.9 |
| **GIRLS** | | | | | | | | | | | | | | |
| **< 2 YEARS** | | | **2-4 YEARS** | | | **5-9 YEARS** | | | **10-17 YEARS** | | | **TOTAL** | | |
| **Disease** | **n** | **%** | **Disease** | **n** | **%** | **Disease** | **n** | **%** | **Disease** | **n** | **%** | **Disease** | **n** | **%** |
| Acute bronchiolitis | 765 | 34.4 | Influenza | 348 | 29.0 | Influenza | 110 | 18,2 | Respiratory failure | 76 | 16.8 | Influenza | 954 | 21.2 |
| Influenza | 433 | 19.7 | Respiratory failure | 178 | 14.8 | Asthma | 97 | 16.0 | Influenza | 59 | 14.7 | Acute bronchiolitis | 786 | 17.5 |
| Respiratory failure | 301 | 13.5 | Other respiratory disorders | 151 | 12.6 | Respiratory failure | 95 | 15.7 | Asthma | 55 | 12.1 | Respiratory failure | 650 | 14.5 |
| Acute bronchitis | 160 | 7.2 | Acute bronchitis | 109 | 9.1 | Other respiratory disorders | 51 | 8.4 | Sepsis | 30 | 6.6 | Other respiratory disorders | 346 | 7.7 |
| Other respiratory disorders | 126 | 5.7 | Asthma | 109 | 9.1 | Acute bronchitis | 31 | 5.1 | Other respiratory disorders | 18 | 4.0 | Acute bronchitis | 311 | 6.9 |
| **BOTH** | | | | | | | | | | | | | | |
| **< 2 YEARS** | | | **2-4 YEARS** | | | **5-9 YEARS** | | | **10-17 YEARS** | | | **TOTAL** | | |
| **Disease** | **n** | **%** | **Disease** | **n** | **%** | **Disease** | **n** | **%** | **Disease** | **n** | **%** | **Disease** | **n** | **%** |
| Acute bronchiolitis | 1702 | 34.2 | Influenza | 675 | 27.0 | Influenza | 221 | 17.0 | Influenza | 139 | 14.8 | Influenza | 1959 | 20,1 |
| Influenza | 914 | 16.0 | Respiratory failure | 349 | 14.1 | Respiratory failure | 212 | 16.4 | Respiratory failure | 134 | 13.2 | Acute bronchiolitis | 1742 | 17.9 |
| Respiratory failure | 702 | 14.1 | Other respiratory disorders | 292 | 11.8 | Asthma | 195 | 15.1 | Asthma | 121 | 12.0 | Respiratory failure | 1397 | 14.3 |
| Acute bronchitis | 349 | 7.0 | Acute bronchitis | 231 | 9.4 | Other respiratory disorders | 135 | 10.5 | Sepsis | 68 | 6.7 | Other respiratory disorders | 764 | 7.8 |
| Other respiratory disorders | 291 | 5,8 | Asthma | 206 | 8.4 | Acute bronchitis | 75 | 5.8 | Other respiratory disorders | 46 | 4.5 | Acute bronchitis | 672 | 6.9 |
